# Cobalt Complex with Thiazole-Based Ligand as New *Pseudomonas aeruginosa* Quorum Quencher, Biofilm Inhibitor and Virulence Attenuator

**DOI:** 10.3390/molecules23061385

**Published:** 2018-06-08

**Authors:** Anabela Borges, Manuel Simões, Tamara R. Todorović, Nenad R. Filipović, Alfonso T. García-Sosa

**Affiliations:** 1 LEPABE, Department of Chemical Engineering, Faculty of Engineering, University of Porto, Rua Dr. Roberto Frias, s/n, Porto 4200-465, Portugal; apborges@fe.up.pt (A.B.); mvs@fe.up.pt (M.S.); 2 Faculty of Chemistry, University of Belgrade, Studentski trg 12–16, Belgrade 11000, Serbia; tamarat@chem.bg.ac.rs; 3 Department of Chemistry and Biochemistry, Faculty of Agriculture, University of Belgrade, Nemanjina 6, Belgrade 11000, Serbia; nenadf.chem@gmail.com; 4 Institute of Chemistry, University of Tartu, Ravila 14a, Tartu 50411, Estonia

**Keywords:** antibacterial resistance, antivirulence/antipathogenic compounds, biofilm prevention, cobalt complex, furvina, pyocyanin, pyoverdine, quorum sensing inhibition, transcriptional activator protein LasR

## Abstract

*Pseudomonas aeruginosa* is one of the most dreaded human pathogens, because of its intrinsic resistance to a number of commonly used antibiotics and ability to form sessile communities (biofilms). Innovative treatment strategies are required and that can rely on the attenuation of the pathogenicity and virulence traits. The interruption of the mechanisms of intercellular communication in bacteria (quorum sensing) is one of such promising strategies. A cobalt coordination compound (Co(**HL**)_2_) synthesized from (*E*)-2-(2-(pyridin-2-ylmethylene)hydrazinyl)-4-(p-tolyl)thiazole (**HL**) is reported herein for the first time to inhibit *P. aeruginosa* 3-oxo-C12-HSL-dependent QS system (LasI/LasR system) and underling phenotypes (biofilm formation and virulence factors). Its interactions with a possible target, the transcriptional activator protein complex LasR-3-oxo-C12-HSL, was studied by molecular modeling with the coordination compound ligand having stronger predicted interactions than those of co-crystallized ligand 3-oxo-C12-HSL, as well as known-binder furvina. Transition metal group 9 coordination compounds may be explored in antipathogenic/antibacterial drug design.

## 1. Introduction

The discovery of antibiotics during the twentieth century is considered one of the most important achievements in the history of medicine, having saved humans from a large number of life-threatening and debilitating diseases [1,2]. Although antibiotics have proven to be powerful drugs for the control of infectious diseases, their extensive and unrestricted use over the last century has imposed selective pressure upon bacteria, leading to the development of resistance. According to the World Health Organization (WHO), antimicrobial resistance is a worldwide problem which is considered a major threat to the treatment of infectious diseases [3]. In addition to this serious problem, the premise that bacteria in biofilms are highly resistant to antimicrobials (100–1000 times more) when compared to their planktonic counterparts presents another obstacle towards the treatment of bacterial infections [4,5]. For some bacteria, working together as a group provides a means to build a defense that is impossible to achieve by planktonic cells [2]. Hence, these observations highlight a strong need to develop therapies that can provide sustainable and long-term effectiveness against bacterial biofilms [2,6].

Typically, therapies with antibiotics are meant to affect bacterial viability, placing a strong selective pressure on bacteria to develop resistance mechanisms. For that reason, much research has been conducted in order to break out of this vicious cycle by interfering with bacterial systems responsible for pathogenicity/virulence [7]. In this context, attention has been focused on strategies for interfering with the quorum sensing (QS) systems of pathogenic bacteria, in order to target their pathogenicity/virulence and to develop new anti-infective therapies. QS is an intercellular communication system mediated by diffusible chemical signal molecules termed autoinducers (AIs) that control gene expression patterns and therefore allows bacteria to synchronize their behavior [8]. In many cases, the responses prompted by QS signals contribute directly to pathogenesis through the production of virulence determinants, such as toxins and proteases. Additionally, QS can contribute to behaviors such as biofilm development that enable bacteria to acquire resistance against antimicrobial compounds [2]. If these efforts to coordinate bacteria behavior are blocked, it is possible that bacterial adaptability will be reduced, facilitating the host immune system to combat the infection and thus reducing the strong selective pressure imposed by conventional antibiotics [9]. Moreover, bacteria will be less able to form organized microbial communities that promote pathogenesis and resistance, such as biofilms [2]. QS inhibitors (QSI) can also improve therapy with antibiotics increasing their effectiveness, favoring the use of lower doses and avoiding the indiscriminate use of broad-spectrum antibiotics [10].

The relationships between QS, virulence regulation and biofilm formation have been most extensively studied in *Pseudomonas aeruginosa*. Therefore, it is not surprising that most of the research on QSI has been centered on this bacterium as a model system [9]. *P. aeruginosa* is a Gram-negative opportunistic pathogen associated with biofilm-related nosocomial infections such as ventilator-associated pneumonia and chronic lung infection in cystic fibrosis patients [11]. Furthermore, it is associated with a high incidence of antibiotic resistance and biofilm formation [12]. *P. aeruginosa* employs at least four different QS circuits to regulate the production of virulence factors and promote biofilm development/maturation, namely the LasRI and RhlRI (LuxRI-type systems), the *Pseudomonas* quinolone signal (Pqs), and the Integrated Quorum Sensing Signal (IQS) [11]. QS genes function in a hierarchical manner with the prominent LasRI system controlling the activity of RhlRI circuit and subsequently the Pqs. The IQS is also strongly controlled by LasRI under rich medium conditions [13]. LasRI and RhlRI systems comprises a transcriptional regulator (LasR and Rh1R, respectively) and its cognate *N*-acyl homoserine lactone (AHL) signal (*N*-(3-oxododecanoyl)-l-homoserine lactone (3-oxo-C12-HSL) and *N*-butyryl-l-homoserine lactone (C4-HSL), respectively), which is synthesized by the AHL synthase (LasI or RhlI, respectively) [14,15]. These systems are responsible for the regulation of virulence factors production, such as protease, exotoxin A, siderophores and pyocyanin, among others. Additionally, the AHL-based QS system triggers a third *P. aeruginosa* quinolone signaling system by producing the AI 2-heptyl-3-hydroxy-4(1H)-quinolone (PQS). This system regulates the expression of virulence factors, biofilm formation, and bacterial motility [12]. As such, new chemical compounds that can disrupt *P. aeruginosa* QS signaling pathways and related mechanisms are welcome for the treatment of important infectious diseases.

**Scheme 1:**
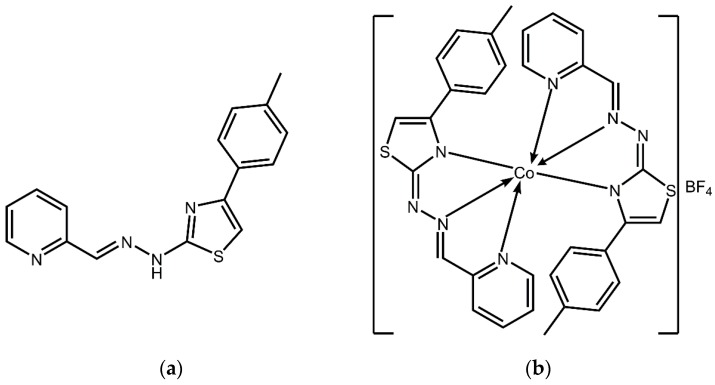
Structures of the hydrazonyl-thiazole based compound, (*E*)-2-(2-(pyridin-2-ylmethylene)hydrazinyl)-4-(p-tolyl)thiazole (**HL**) (**a**) and its Co(III) complex; Co(**HL**)_2_ (**b**).

The thiazole ring is one of the most important scaffolds in medicinal chemistry [16]. In our previous work, a hydrazonyl-thiazole-based compound, (*E*)-2-(2-(pyridin-2-ylmethylene)hydrazinyl)-4-(p-tolyl)thiazole (**HL**, Scheme 1), was synthesized in order to test its activity against cancer cells. Due to poor solubility in culture medium, the activity of **HL** could not be tested, so its cobalt(III) complex ([Co(**HL**)_2_]BF_4_, Scheme 1) was prepared. Our results revealed that Co(**HL**)_2_ showed stronger activity on human mammary adenocarcinoma (MCF-7) cancer cells than cisplatin [17]. Additionally, the antibacterial activity of Co(**HL**)_2_ was tested by the disc diffusion method on several Gram-positive (*Staphylococcus aureus*, *Clostridium sporogenes*, *Bacillus subtilis,* and *Kocuria rhizophila*) and Gram-negative (*Proteus hauseri*, *P. aeruginosa*, *Escherichia coli*, and *Salmonella enterica*) strains. The results obtained showed that Co(**HL**)_2_ possesses antibacterial activity comparable to antibiotic amikacin [18]. The latter result encouraged us to test the activity of Co(**HL**)_2_ on QS inhibition and QS-dependent phenotypes, namely biofilm development and virulence factors production.

In the present work, Co(**HL**)_2_ was investigated as a *P. aeruginosa* LasI/LasR QS/biofilm formation inhibitor and virulence attenuator (pyocyanin and pyoverdine inhibition), as well as its interactions with a possible target, the transcriptional activator protein LasR, by molecular docking simulations. To the best of our knowledge, this work reports the first investigation of the activity of a metal complex on *P. aeruginosa* signaling pathways and its biofilms. Interestingly, the results show that Co(**HL**)_2_ can inhibit this type of cell-to-cell communications system (LasI/LasR) and underlying phenotypes and that this complex aids the **HL** to have a stronger interaction with the target protein than the known inhibitors (e.g., furvina).

## 2. Results and Discussion

### 2.1. Cordination Complex Stability Analysis

Before evaluation of antimicrobial activity, the aqueous solution behavior of the complex Co(**HL**)_2_ with respect to hydrolysis was studied in dimethyl sulfoxide/water (DMSO/H_2_O, 6% vv^−1^) at 298 K over 24 h by ultraviolet/visible (UV/Vis) spectroscopy. The complex was quite stable, as can be seen from the electronic absorption spectra (See Supplementary Materials, Figure S1). Only a small portion of the complex (~5%) was hydrolysed during 24 h period.

### 2.2. Antimicrobial Activity of Co(**HL**)_2_

In order to know the inhibitory and bactericidal activities of Co(**HL**)_2_, as well as to choose the suitable concentrations for QS inhibition assays, the minimum inhibitory and bactericidal concentrations (MIC and MBC) were determined. MIC and MBC values obtained for Co(**HL**)_2_ against *P. aeruginosa* PA14 wild-type and biosensor *P. aeruginosa* PA14-R3 are presented in Table 1. Both strains exhibited the same MIC values of 800 µg mL^−1^. No bactericidal activity was detected below 1000 µg mL^−1^.

### 2.3. Co(**HL**)_2_ Mediated Inhibition of the 3-oxo-C12-HSL-Dependent QS System of P. aeruginosa

The *P. aeruginosa* PA14/PA14-R3 co-cultivation system was used to screen the global effect of Co(**HL**)_2_ on the *P. aeruginosa* 3-oxo-C12-HSL-dependent QS system. Due to this method being revealed to be sensitive to DMSO, concentrations of this solvent equal to or lower than 6% (vv^−1^) of the final volume of cell suspension were applied.

According to Imperi et al. [9], the criteria used for selection of hit compounds were: at least 50% of relative bioluminescence emission and a maximum of 20% in the reduction of cell growth with respect to the negative controls (cells with DMSO at 6%). The latter criterion was aimed at avoiding any unspecific effect of impaired growth on the QS response.

**Figure 1:**
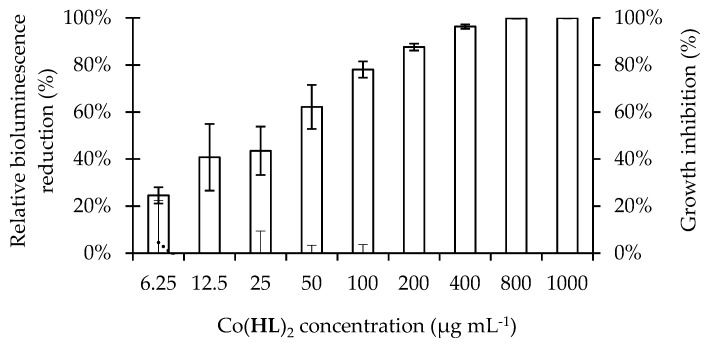
Effect of increasing concentrations of Co(**HL**)_2_ (6.25 to 1000 µg mL^−1^) on *P. aeruginosa* 3-oxo-C12-HSL-dependent QS system (bars) in general and on growth inhibition (dashed line) (based on the co-culture of wild-type/biosensor). Bioluminescence emissions were normalized to the cell density of the bacterial culture and expressed as percentages with respect to untreated controls (cells + DMSO at 6%; relative bioluminescence). Mean values ± standard deviations for at least three replicates are illustrated.

The results of the QS inhibition screening, performed using Co(**HL**)_2_ in a range of different concentrations (6.25 to 1000 µg mL^−1^), are depicted in Figure 1. Co(**HL**)_2_ interfered with *P. aeruginosa* 3-oxo-C12-HSL-dependent QS system and its effect was dose-dependent (25 to 100%). Total bioluminescence reduction was achieved with the higher concentrations evaluated (800 and 1000 µg mL^−1^). This initial screening assay allowed the identification of Co(**HL**)_2_ as a putative QSI that inhibited the QS response of the *P. aeruginosa* PA14/PA14-R3 co-cultivation system, without affecting bacterial growth (Figure 1, secondary *y*-axis). This is in accordance with the premise that any compound able to interfere with QS without affecting cell growth can be considered a promising inhibitor [19]. Indeed, the selection of an effective QSI is based on its specificity for a given QS regulator with no or little adverse effects on bacteria or host [20].

**Figure 2:**
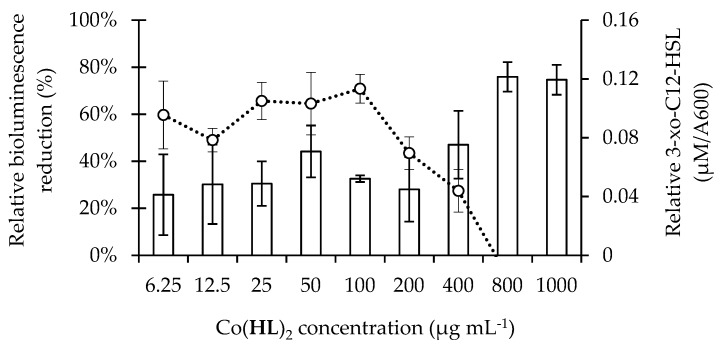
Effect of increasing concentrations of Co(**HL**)_2_ (6.25 to 1000 µg mL^−1^) on the production of 3-oxo-C12-HSL by *P. aeruginosa* PA14 (wild-type strain) (bars) and quantification of the 3-oxo-C12-HSL produced levels (dashed line). Bioluminescence emissions were normalized to the cell density of the bacterial culture and expressed as percentages with respect to untreated controls (cells + DMSO at 6%; relative bioluminescence). 3-oxo-C12-HSL levels were expressed as percentages with respect to untreated controls (cells + DMSO at 6%; relative 3-oxo-C12-HSL). Mean values ± standard deviations for at least three replicates are illustrated.

**Figure 3:**
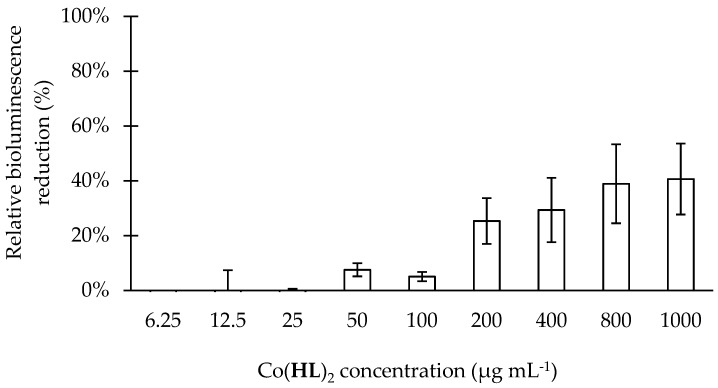
Effect of increasing concentrations of Co(**HL**)_2_ (6.25 to 1000 µg mL^−1^) on the detection of 3-oxo-C12-HSL by *P. aeruginosa* PA14-R3 (biosensor strain). Bioluminescence emissions were normalized to the cell density of the bacterial culture and expressed as percentages with respect to untreated controls (cells + DMSO at 6%; relative bioluminescence). Mean values ± standard deviations for at least three replicates are illustrated.

To further study the effect of Co(**HL**)_2_ on the *P. aeruginosa* 3-oxo-C12-HSL-dependent QS system, additional assays were performed in order to find out if this compound interfered with the production and/or detection of the 3-oxo-C12-HSL AI (Figure 2 and Figure 3). Figure 2 shows the effect of Co(**HL**)_2_ on the production of 3-oxo-C12-HSL. Bioluminescence reductions between 26% and 76% and continuous decrease of the levels of 3-oxo-C12-HSL produced were found. 

Relative to the effect of Co(**HL**)_2_ on the AI detection (Figure 3), the data shows a dose-dependent activity with a maxiumum reduction of 41%. Co(**HL**)_2_ demonstrated ability to interfere with both production and detection mechanisms, being this effect less pronounced on the 3-oxo-C12-HSL detection.

### 2.4. Effect of Co(**HL**)_2_ on the Prevention of Biofilm Formation

**Figure 4:**
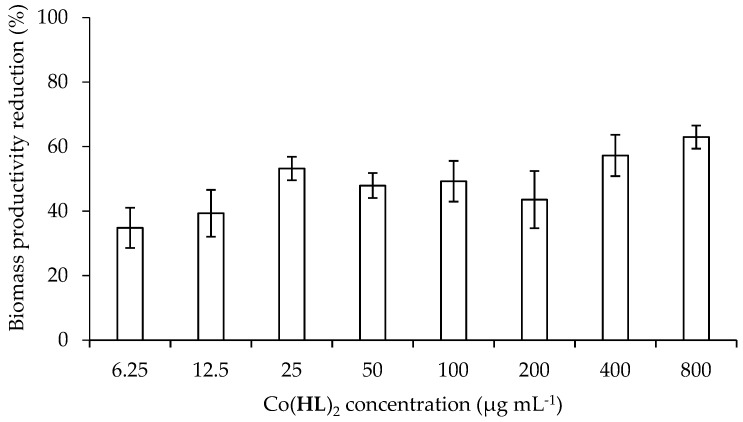
Preventive action of Co(**HL**)_2_ at MIC (800 µg mL^−1^) and sub-inhibitory concentrations (6.25 to 400 µg mL^−1^) on biomass productivity of *P. aeruginosa* PA14 (wild-type strain) biofilm. Mean values ± standard deviations for at least three replicates are illustrated.

*P. aeruginosa* is responsible for chronic infections due to biofilm formation ability. It is known that QS is an important event that is linked with the different steps of bacterial biofilm development (initial formation, maturation, and dispersal) and dynamic (heterogeneity, architecture, stress resistance, maintenance, and sloughing). The blockage of QS pathways can lead to formation of biofilms more unstructured/cohesive and susceptible to host defenses and chemical agents favouring the use of low doses of antimicrobials and leading to an easier removal/eradication [21,22]. Numerous studies using flow cell chambers for biofilm formation, together with confocal scanner electron microscopy observations, have shown that a proficient QS system is essential for optimal biofilm development [23]. The first evidence that QS influences biofilm formation in *P. aeruginosa* was the finding that a LasI mutant produces a thinner biofilm that was more susceptible to disruption by detergents [24]. In this context, the preventive effect of putative QSI Co(**HL**)_2_ at the MIC (800 µg mL^−1^) and sub-inhibitory concentrations (6.25 to 400 µg mL^−1^) on *P. aeruginosa* PA14 biomass production was studied (Figure 4). It was observed that Co(**HL**)_2_ significantly decreased the ability of *P. aeruginosa* to establish biofilms (*p* < 0.05). Total biofilm mass productivity reduction was not achieved. However, Co(**HL**)_2_ promoted biomass reductions from 35 to 63%. No effect was observed on the metabolic activity of the biofilm cells (data not shown). It is known that any compound able to interfere with QS without affecting cell growth can be considered a promising inhibitor [19].

### 2.5. Effect of Co(**HL**)_2_ on the Production of Pyocyanin and Pyoverdine Virulence Factors

*P. aeruginosa* produces a wide array of virulence factors and evades the immune system by a great variety of adaptive mechanisms. Treatment of *P. aeruginosa* infection is difficult to achieve due to the problem of multidrug resistance and to the production of several virulence factors including pyocyanin, siderophores, and proteases, among others, that contribute to its pathogenesis [24]. The production of many of the key virulence factors is controlled by QS. Thus, the disruption of this process by chemical interference is becoming a topic of increasing interest in the pharmaceutical industry and academia [25].

Most studies of QS in *P. aeruginosa* have been focused on its role in pathogenicity. In this context, the effect of QSI on the inhibition of virulence factor production controlled by QS such as pyocyanin and pyoverdine was also studied. Pyocyanin, a redox-active small molecule, is one key virulence factor produced by *P. aeruginosa* at high cell density in response to the *Las* and *Rhl* AHL signal. In addition to its role as a terminal signal in the QS pathway, this phenazine derivative has the ability to maintain *P. aeruginosa* redox balance, mainly under low oxygen conditions, and also protect it from reactive oxygen species [26]. In addition to this, pyocyanin induces neutrophil apoptosis, inflammatory response, and neutrophil-mediated tissue damage [27].

**Figure 5:**
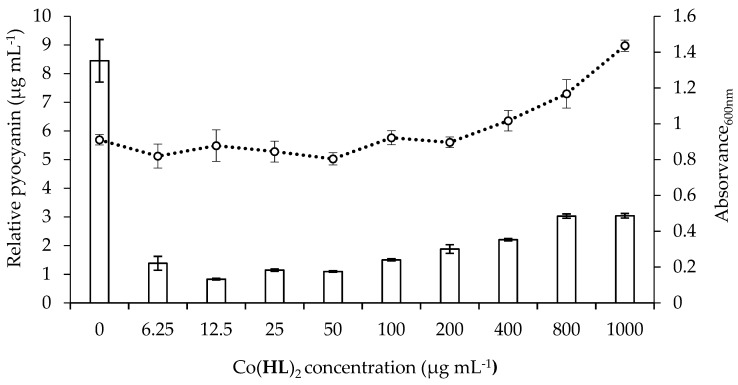
Influence of Co(**HL**)_2_ on pyocyanin production (bars) and on cell growth (A_600nm_) (dashed line) of *P. aeruginosa* PA14 (wild-type strain) as a function of the different concentrations (6.25 to 1000 µg mL^−1^). The levels of pyocyanin were measured in cell-free supernatants from cultures of *P. aeruginosa* PA14 (wild-type strain). The total amount of protein was calculated (μg mL^−1^), normalized per cell density (A_600nm_ values) and expressed as relative pyoverdine production. Mean values ± standard deviations for at least three replicates are illustrated.

The efficacy of Co(**HL**)_2_ at reducing pyocyanin levels was calculated with respect to cell suspension without treatment. Co(**HL**)_2_ demonstrated ability to considerably reduce the amount of pyocyanin produced as can be observed in Figure 5. A maximum reduction of ~90% was acquired at 12.5 µg mL^−1^. No significant growth inhibition was observed.

*P. aeruginosa* also secretes two types of siderophores, pyoverdine and pyochelin, which are high-affinity and lower-affinity iron-chelating compounds, respectively, with an important role in the incorporation of iron in proteins. These iron-chelating molecules compete with mammalian cells for iron, and when successful sequestration occurs, they starve the host tissues [28]. These molecules are also involved in the regulation of the expression of genes related with the production of virulence factors (e.g., exotoxin A, an endoprotease, and pyoverdine itself) which are major contributors to *P. aeruginosa* pathogenicity [28]. Any factor influencing the siderophore secretion by *P. aeruginosa* would greatly influence the efficacy of this opportunistic pathogen in promoting disease, since they are important for both bacterial virulence and biofilm development.

**Figure 6:**
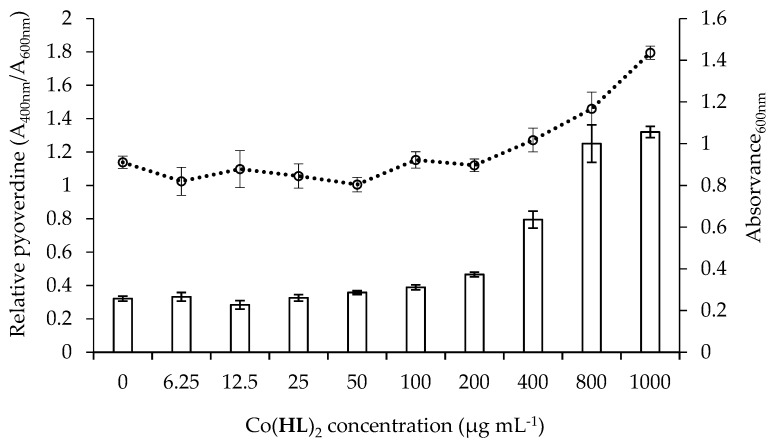
Influence of Co(**HL**)_2_ on pyoverdine production (bars) and on cell growth (A_600nm_) (dashed line) of *P. aeruginosa* PA14 (wild-type strain) as a function of the different concentrations (6.25 to 1000 µg mL^−1^). The levels of pyoverdine were measured in cell-free supernatants from cultures of *P. aeruginosa* PA14 (wild-type strain), normalized per cell density (A_600nm_ values) and expressed as relative pyoverdine production. Mean values ± standard deviations for at least three replicates are illustrated.

To address the question whether Co(**HL**)_2_ alters the production of pyoverdine by *P. aeruginosa*, the absorbance values of cell-free supernatants from cultures of the *P. aeruginosa* wild-type strain (PA14) were assessed in the presence of this compound and compared with negative control (cells + DMSO at 6%). Apparently, Co(**HL**)_2_ had no effect on the pyoverdine production (Figure 6).

### 2.6. Molecular Docking Analysis

Given that the transcriptional activator protein complex LasR-3-oxo-C12-HSL can coordinate the expression of target genes with cell density, including many genes that encode virulence factors for *P. aeruginosa* [28], it may be a target for QSI, and so docking was performed on this protein target. Results from docking showed that the strongest interaction score came from the coordination ligand **HL**, rather than from the whole complex (Co(**HL**)_2_) (Table 2). In fact, **HL** formed the strongest interaction, when compared with the known ligand furvina, as well as the co-crystallized ligand 3-oxo-C12-HSL (HET-ID OHN) and Co(**HL**)_2_.

Indeed, neither Quantum-Polarized Ligand Docking (QPLD) nor Autodock4 could find a strong interaction or good binding pose for the whole cobalt complex in the binding site, given its large size. On the other hand, all of the docking programs and scoring functions were in consensus that the strength of the interaction to the protein was in the order: **HL** > 3-oxo-C12-HSL > furvina > Co(**HL**)_2_. Based on these results, it may be possible that the coordination complex delivers the ligands to the site of action of the protein, where they dissociate and form strong protein-ligand complexes with the transcriptional activator protein LasR. Both functions, delivery and protein-binding, are probably very important to the ligand′s efficacy [29,30]. This is of additional importance considering that the ligand **HL** is less soluble in uncoordinated form as opposed to coordinated cobalt complex form.

**Figure 7:**
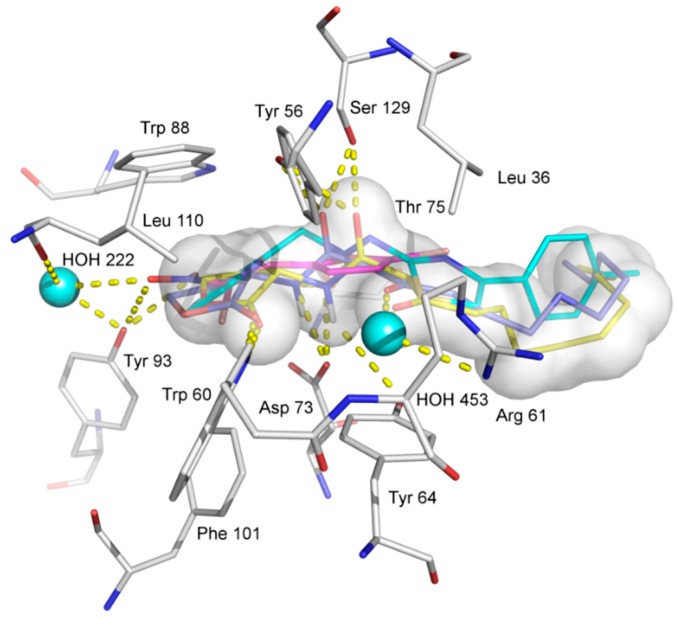
Docked ligands **HL** in cyan, docked known inhibitor furvina in magenta, docked known binder 3-oxo-C12-HSL in slate (HET-ID OHN), co-crystallized (X-ray) known binder 3-oxo-C12-HSL in yellow, in the binding site of transcriptional activator protein LasR (3IX3) in white, hydrogen bonds as dashes, sulphur in yellow, oxygen in red, nitrogen in blue, and halogen in salmon.

The docked ligands, as well as co-crystallized ligand are shown in the binding site of LasR in Figure 7. A good overlap between the ligands can be seen, as well as hydrogen bonds and hydrophobic interactions made with the protein and with bridging water molecules HOH 222 and 453.

The results offer a possible route for cobalt coordination complexes as *P. aeruginosa* anti-QS compounds, biofilm inhibitors, and virulence attenuators. Such possibilities expand the use of group 9 transition metal complexes as biomolecular ligands [31,32] and their possible use in drug design.

## 3. Materials and Methods

### 3.1. Reagents, Apparatus, and Synthesis

Thiosemicarbazide (99%), 2-pyridinecarboxaldehyde (99%), and 2-bromo-4'- methylacetophenone were obtained from Acros Organics (BVBA, Geel, Belgium). Cobalt tetrafluoroborate hexahydrate was obtained from Aldrich (Sigma-Aldrich Chemie GmbH, Steinheim, Germany). All solvents (reagent grade) were obtained from commercial suppliers and used without purification. Elemental analyses (C, H and N) were performed by the standard micromethods using the ELEMENTAR Vario ELIII C.H.N.S=O analyzer. Infrared spectra were recorded on a Thermo Scientific Nicolet 6700 FT-IR spectrophotometer by the attenuated total reflection technique from 4000–400 cm^−1^. Molar conductivity measurement was performed at ambient temperature on a Crison Multimeter MM41. ^1^H and ^13^C NMR spectra were performed on Bruker Avance 500, equipped with a broad-band direct probe. All spectra were measured at 298 K. Co(**HL**)_2_ was prepared by reaction of **HL** and Co(BF_4_)_2_·6H_2_O in MeOH as emerald colored single crystals as described previously [17]. IR and NMR spectroscopy data, as well as molar conductivity measurements and results of elemental analysis for Co(**HL**)_2_ are in good agreement with the data previously published [17]. The UV/Vis spectra during a 24 h period were recorded with a GBC Scientific Cintra 6 UV/Vis spectrophotometer (250–800 nm) with sample dissolved in DMSO and diluted with water such that the final DMSO content was 6% (vv^−1^).

### 3.2. Bacterial Strains, Growth Conditions, and Solutions

To seek for QS inhibition activity, two bacterial strains, *P. aeruginosa* PA14 wild-type and *P. aeruginosa* PA14-R3 biosensor, were used [33]. The biosensor is a mutant of the wild-type strain that is not capable of producing its own AI (3-oxo-C12-HSL), but is able to detect/respond to exogenous AI with bioluminescence emission [33]. In the *P. aeruginosa* PA14-R3 biosensor a transcriptional fusion between the LasR-dependent *rsaL* promoter and the *luxCDABE* operon was chromosomally integrated at the *attB* neutral site of the chromosome. Additionally, the *lasI* gene encoding 3-oxo-C12-HSL synthase was inactivated by transposon insertion [34]. These strains were kindly provided by Professor Livia Leoni (University Roma Tre, Rome, Italy). Bacterial cultures were grown aerobically overnight (≈ 16 h) in Luria–Bertani broth (LBB; Liofilchem, Roseto degli Abruzzi, Italy) at 37 °C in a shaking incubator (150 rpm) (AGITORB 200, Aralab, Rio de Mouro, Portugal), prior to each experiment. Co(**HL**)_2_ stock solution was prepared in 100% (vv^−1^) dimethyl sulfoxide (DMSO; Fisher Scientific, Loughborough, UK) under sterile conditions. Serial dilutions were prepared when needed and the percentage of DMSO never exceeded 6% (vv^−1^) of the final volume of cell suspension. Negative controls sets containing cell suspensions with DMSO and without Co(**HL**)_2_ were used. Also, a cell suspension with (Z-)-4-bromo-5-(bromomethylene)-2(5H)-fu (Furanone C-30–FC30) was used as a positive control (due to its known *P. aeruginosa* QS inhibition potential) [9,35] for QS inhibition assays and the known QSI furvina was used as positive control for other assays (biofilm prevention, virulence factors and molecular modeling). All assays were performed in triplicate with a minimum of three repeats.

### 3.3. Minimum Inhibitory and Bactericidal Concentrations (MIC and MBC)

The MIC of Co(**HL**)_2_ was determined by the microdilution method [36]. Briefly, bacterial overnight (≈16 h) cultures were taken and adjusted to an absorbance value of 0.1 ± 0.02 (A_600nm_) (7.4 × 10^7^ CFU (colony-forming units) mL^−1^; determined by CFU counts in solid medium). Then, 96-well clear bottomed polystyrene (PS) microtiter plates (Orange Scientific, Braine-l’Alleud，Belgium) were filled with 180 µL of cells and 20 µL of Co(**HL**)_2_ at different concentrations (6.25 to 1000 µg mL^−1^), and incubated at 37 °C and 150 rpm for 24 h. Absorbance measurements were performed before (t = 0 h) and after (t = 24 h) the incubation period using a microplate reader (FLUOstar Omega; BMG LABTECH, Ortenberg, Germany). The MIC was recorded as the lowest concentration of compound which showed no difference between the absorbance values measured at both distinct times (no bacterial growth is detected). The drop method was applied for MBC determination, which consists of directly removing a volume of 10 µL from the wells containing Co(**HL**)_2_ concentrations equal to and above the MIC, and plates out on LB agar (LBA; Merck, Darmstadt, Germany). Plates were incubated at 37 °C for 24 h and the growth was visually inspected. The MBC was recorded as the lowest concentration of compound in which total growth inhibition was observed [35].

### 3.4. High-Throughput QS Inhibition Screening

The ability of Co(**HL**)_2_ to interfere with the QS response of *P. aeruginosa* was evaluated using a high-throughput QS inhibition screening system based on a co-cultivation assay (*P. aeruginosa* PA14-R3 biosensor and *P. aeruginosa* PA14 wild-type) [33]. For this, *P. aeruginosa* PA14 wild-type and *P. aeruginosa* PA14-R3 biosensor were grown overnight at 37 °C on LBA plates. Then, some colonies were taken from the plate surfaces and diluted in LBB to an absorbance (A_600nm_) values of 0.045 (5.80 × 10^7^ CFU mL^−1^) and 0.015 (2.15 × 10^7^ CFU mL^−1^) for *P. aeruginosa* PA14-R3 biosensor and *P. aeruginosa* PA14 wild-type, respectively (3:1 reporter/wild-type ratio). Black 96-well opaque bottomed PS microtiter plates and 96-well clear bottomed PS microtiter plates were filled with co-culture (180 µL) and Co(**HL**)_2_ (20 µL) in a range of different concentrations (6.25 to 1000 µg mL^−1^). The light counts per second (LCPS) and A_600nm_ were measured after 4 h of growth using a microplate reader. Luminescence values were normalized, dividing LCPS values by the A_600nm_ values.

#### 3.4.1. Autoinducer Synthesis Assay

The effect of Co(**HL**)_2_ on the production of the AI 3-oxo-C12-HSL was evaluated according to Massai et al. [33]. Firstly, *P. aeruginosa* PA14 was grown for 24 h at 37 °C on LBA. After that, bacteria were scrapped from the plate surfaces and diluted in LBB to an absorbance (A_600nm_) of 0.05 (5.85 × 10^7^ CFU mL^−1^) and incubated overnight (≈16 h) at 37 °C and 150 rpm, in the absence and presence of Co(**HL**)_2_ in a range of different concentrations (6.25 to 1000 µg mL^−1^). Afterwards, the growth of bacterial suspensions was examined and the remaining volume centrifuged for 20 min at 3772 g. Black 96-well opaque bottomed PS microtiter plates and 96-well clear bottomed PS microtiter plates were filled with cell free supernatant (20 µL) and PA14-R3 cell suspension (180 µL), prepared as previously described for PA14 (A_600nm_ = 0.045). After 4 h of incubation at the temperature and agitation conditions indicated above, the LCPS and A_600nm_ were measured using a microplate reader. Luminescence values were normalized dividing LCPS values by the A_600nm_ values. A calibration curve was generated by growing *P. aeruginosa* PA14-R3 in the presence of increasing concentrations of synthetic 3-oxo-C12-HSL, to calculate the concentration of 3-oxo-C12-HSL in each culture supernatant (See Supplementary Materials, Figure S2).

#### 3.4.2. Autoinducer Response Assay

To evaluate the influence of Co(**HL**)_2_ in the detection of the AI 3-oxo-C12-HSL a similar protocol to the AI synthesis was used. For this, *P. aeruginosa* PA14-R3 was grown for 24 h at 37 °C in LBA plates. Then, the bacteria were scrapped from the plate surface and diluted in LBB to an absorbance (A_600nm_) of 0.05 (5.85 × 10^7^ CFU mL^−1^) and overnight incubated at 37 °C and 150 rpm in the absence and presence of Co(**HL**)_2_ in a range of different concentrations (6.25 to 1000 µg mL^−1^). After the incubation period the bacterial growth was inspected and the cell suspensions adjusted to an absorbance (A_600nm_) of 0.045. Black 96-well opaque bottomed PS microtiter plates and 96-well clear bottomed PS microtiter plates were filled with PA14-R3 cellular suspension (180 µL) and synthetic 3-oxo-C12-HSL (20 µL) at concentration of 25 µM. After 4 h of incubation at the temperature and agitation conditions indicated above, the LCPS and A_600nm_ were measured using a microplate reader. Luminescence values were normalized dividing LCPS values by the A_600nm_ values.

### 3.5. Biofilm Prevention Assays

Biofilms were developed according to the modified microtiter plate assay proposed by Stepanović et al. (2000). Briefly, 96-well clear bottomed PS microtiter plates were filled with 180 μL of suspension of *P. aeruginosa* PA14 wild-type (A_600 nm_ = 0.04 (4.9 × 10^7^ CFU mL^−1^)) and 20 μl of Co(**HL**)_2_ at the MIC (800 μg mL^−1^) and the sub-inhibitory concentrations (6.25 to 400 μg mL^−1^). The plates were incubated at 37 °C and 150 rpm for 24 h. After the incubation period, the content of each well was discarded and washed twice with saline solution (NaCl, 0.85%). The plates were analyzed in terms of biomass formation by crystal violet (CV; Merck, Darmstadt, Germany) and alamar blue staining’s [37] and the results were expressed as percentages of biomass productivity and metabolic activity reduction [38].

### 3.6. Virulence Factors Assays

#### 3.6.1. Pyocyanin Production

Pyocyanin was extracted from PA14 wild-type culture supernatants and measured as previously described by [39]. Briefly, PA14 wild-type suspension (A_600nm_ = 0.05) (5.85 × 10^7^ CFU mL^−1^) was grown in the presence of Co(**HL**)_2_ in a range of different concentrations (6.25 to 1000 µg mL^−1^), at 37 °C and 150 rpm for 16 h. After incubation, the absorbance of the grown cultures were measured (A_600nm_) and the cells were then harvested by centrifugation (15 min at 12,000 g), and the culture supernatant recovered. Pyocyanin was extracted by mixing chloroform with culture supernatant (3:5 chloroform/supernatant ratio). Then, the chloroform layer (lower blue layer) was transferred to a fresh tube and mixed with 1 mL of 0.2 M hydrochloric acid (HCl; Fisher Chemical, Merelbeke, Belgium). The A_520nm_ of the resulting solution (upper red/pink layer) was measured to determine the amount of extracted pyocyanin. The amount of pyocyanin, in μg mL^−1^, was calculated by multiplication of the A_520nm_ values for 17.072 and was normalized dividing A_520nm_ values by the A_600nm_ values [(A_520nm_/A_600nm_) × 17.072].

#### 3.6.2. Pyoverdine Production

Pyoverdine production was measured according to a spectrophotometric method previously described by Höfte et al. [40], with some modifications. Briefly, the concentration of pyoverdine in the culture supernatant was measured directly at 400 nm. Relative pyoverdine production was estimated by dividing A_400nm_ values by the A_600nm_ values of the cultures [41].

### 3.7. Molecular Modeling of Co(**HL**)_2_ with LasR Receptor

Ligand structures were based on the X-ray crystal structure [17]. The protein crystal structure of the transcriptional activator protein LasR-3-oxo-C12-HSL complex was downloaded from the Protein Databank (1ix3) [Protein Data Bank. Research Collaboratory for Structural Bioinformatics. http://www.pdb.org/pdb/home/home.do (accessed March 2018)]. The protein and ligand structures were preprocessed with Maestro [Schrödinger, LLC: New York, 2017], including determining protonation states and adding hydrogen atoms, and then docking was performed on the LasR protein using compound Co(**HL**)_2_, the coordination ligand **HL** on its own, as well as the co-crystallized ligand 3-oxo-C12-HSL, and the known inhibitor furvina (2-Bromo-5-(2-bromo-2-nitrovinyl)furan, G1). Docking used three programs: (1) Quantum-Polarized Ligand Docking (QPLD) using Glide XP scoring function in Schrödinger [Schrödinger, LLC: New York, 2017], using default parameters in addition to no Epik penalties; (2) the Autodock4 docking program and scoring function [42] with settings intelec (calculate internal electrostatics) ON, tran0 random initial coordinates, axisangle0 random initial orientation, dihe0 random initial dihedrals, tstep 2.0 Å translation step, qstep 50.0 degrees quaternion step, dstep 50.0 degrees torsion step, torsdof 2 torsional degrees of freedom, rmstol 2.0 Å cluster_tolerance, extnrg 1000.0 external grid energy, e0max 0.0 max initial energy, 10000 max number of retries, ga_pop_size 250 number of individuals in population, ga_num_evals 20000000 maximum number of energy evaluations, ga_num_generations 27000 maximum number of generations, ga_elitism 1 number of top individuals to survive to next generation, ga_mutation_rate 0.02, ga_crossover_rate 0.8, ga_window_size 10, ga_cauchy_alpha 0.0 Alpha parameter of Cauchy distribution, ga_cauchy_beta 1.0 Beta parameter Cauchy distribution, genetic algorithm, sw_max_its 300 iterations of Solis & Wets local search, sw_max_succ 4 consecutive successes before changing rho, sw_max_fail 4 consecutive failures before changing rho, sw_rho 1.0 size of local search space to sample, sw_lb_rho 0.01 lower bound on rho, ls_search_freq 0.06 probability of performing local search on individual, unbound_model extended state of unbound ligand; 3) the Autodock Vina docking program and scoring function [43] with settings: size_x = 25, size_y = 25, size_z = 25, num_modes = 9, energy_range = 1, exhaustiveness = 64, cpu = 1. Checks were performed as before [44,45].

### 3.8. Statistical Analysis

The data were analyzed using paired sample *t*-test from the SPSS (Statistical Package for the Social Sciences) program (IBM^®^ SPSS^®^ Statistics, Lisbon, Portugal), version 20.0. The mean and standard deviation (SD) within samples were calculated for all experiments. All tests were done in triplicate with three independent repeats for each condition tested. The statistical calculations were based on a confidence level of ≥95% (*p* < 0.05 was considered statistically significant).

## 4. Conclusions

For the first time, a coordination complex is reported to inhibit *P. aeruginosa* LasR QS system as well as their biofilms and QS regulated virulence factors. The results of molecular docking on transcriptional activator protein LasR suggest that the possible mechanism involves the coordination complex not directly binding the protein. Instead, the ligands from the coordination complex can be responsible for binding to the transcriptional activator protein LasR target. The ligand from this cobalt coordination compound, **HL**, has stronger predicted interactions than those of the known-binder and co-crystallized ligand 3-oxo-C12-HSL, as well as those of another known-binder, furvina. It is interesting to note that the ligand **HL** is also a chelator, as is the case of the QS compound pyoverdine produced by *P. aeruginosa*. In addition, the ligand **HL** is more soluble in the cobalt coordination form than uncomplexed, so cobalt complexation may aid in increasing its solubility and delivery to the protein site of action. Transition metal group 9 coordination compounds can thus be explored as QS and biofilm inhibitors, as well as their possible use in drug design and delivery.

## Figures and Tables

**Table 1:** Minimum inhibitory (MIC) and minimum bactericidal (MBC) concentrations values of Co(**HL**)_2_ against *P. aeruginosa* PA14 wild-type and biosensor PA14-R3.

Compound	*P. aeruginosa* PA14		*P. aeruginosa* PA14-R3	
	MIC (µg mL^−1^)	MBC (µg mL^−1^)	MIC (µg mL^−1^)	MBC (µg mL^−1^)
Co(HL)_2_	800	>1000 ^1^	800	>1000 ^1^

^1^ MBC not found, it is higher than the maximum concentration tested (1000 µg mL^−1^).

**Table 2:** Docking interaction scores (kcal/mol) with transcriptional activator protein LasR.

Compound	QPLD	Autodock4	Vina
Co(**HL**)_2_	-	+51.94	−4.0
**HL**	−8.86	−8.41	−10.1
Furvina	−4.71	−5.74	−7.0
3-oxo-C12-HSL	−8.60	−7.39	−8.7

## Supplementary Materials

Supplementary Materials are available online.
