# Supplementary material for: Cobalt Complex with Thiazole-Based Ligand as New Pseudomonas aeruginosa Quorum Quencher, Biofilm Inhibitor and Virulence Attenuator
Source: Molecules. 2018 Jun 8;23(6):1385. doi: 10.3390/molecules23061385 (PMC6099793; doi:10.3390/molecules23061385)
Supplement: Supplementary file 1 [file molecules-23-01385-s001.pdf]

Cobalt complex with thiazole-based ligand as new *Pseudomonas aeruginosa* quorum quencher, biofilm inhibitor, and virulence attenuator

Anabela Borges, Manuel Simões, Tamara R. Todorović, Nenad R. Filipović and Alfonso T. García-Sosa*

t.alfonso@gmail.com

Contents

Supplementary Figure S1 2
Supplementary Figure S2 3

Supplementary Figure S1

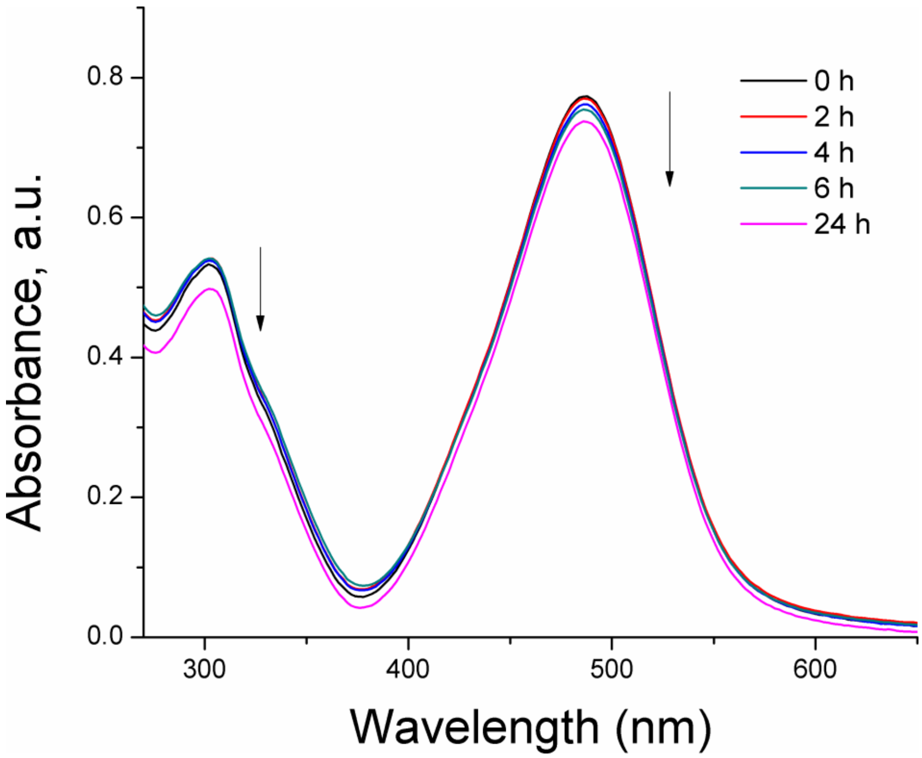

Caption. The UV-Vis spectroscopy spectra for the complex Co(**HL**)$_2$ in DMSO/H_2O 6 : 100 (v/v) at 298 K. First measurement (black), after 24 h (pink).

Supplementary Figure S2

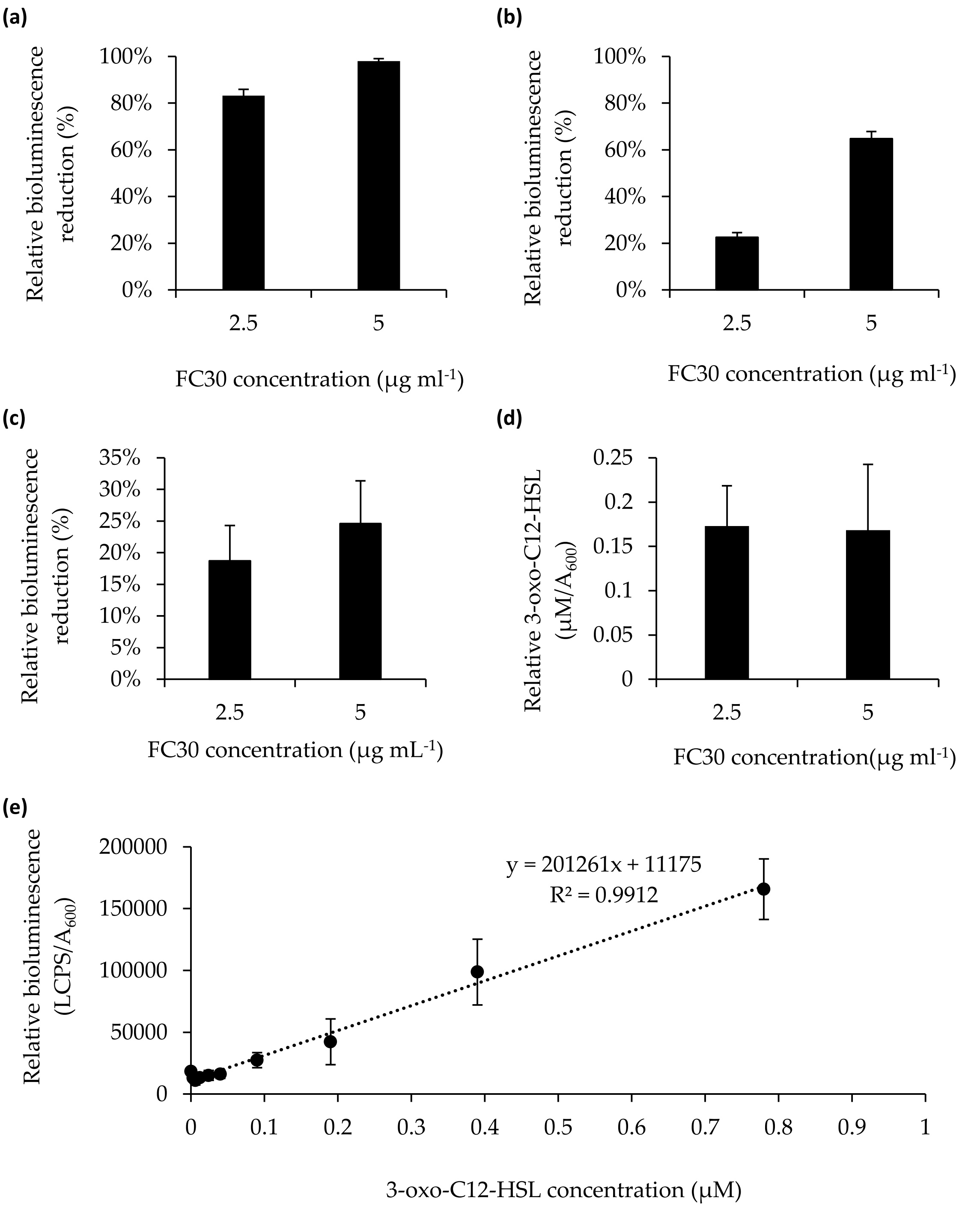

Figure S1. Positive controls with furanone C-30 (FC30) (known QS inhibitor) at 2.5 and 5 μg mL^{-1}. Interference of FC30 with *P. aeruginosa* 3-oxo-C12-HSL-based QS system (a). Effect of FC30 on both 3-oxo-C12-HSL detection by PA14-R3 (b) and 3-oxo-C12-HSL production by PA14 wild-type (c). The concentration of 3-oxo-C12-HSL present in culture supernatant of PA14 wild-type strain previously

exposed to FC30 (2.5 and 5 μg mL^{-1}) was detected by PA14-R3 biosensor and quantitatively determined (d) based in an equation derived from calibration curve (e).
